# Whole exome sequencing diagnosis of inborn errors of metabolism and other disorders in United Arab Emirates

**DOI:** 10.1186/s13023-016-0474-3

**Published:** 2016-07-08

**Authors:** Aisha Al-Shamsi, Jozef L. Hertecant, Abdul-Kader Souid, Fatma A. Al-Jasmi

**Affiliations:** Department of Pediatrics, Tawam Hospital, Al-Ain, UAE; Department of Pediatrics, United Arab Emirates University, P.O. Box 17666, Al-Ain, UAE

**Keywords:** Whole exome sequencing, Mutations, Variants, Inborn errors of metabolism, UAE

## Abstract

**Background:**

This study reports on the use of whole exome sequencing (WES) to diagnose children with inborn errors of metabolism and other disorders in United Arab Emirates.

**Methods:**

From January 2012 to December 2014, 85 patients (46 % females) were seen in the metabolic center at Tawam Hospital (Abu Dhabi) and WES testing was requested because definitive diagnoses were not reached by conventional methods.

**Results:**

Eighty (93 %) patients were <18 years old and 44 (52 %) were <5 years. Sixty-eight (80 %) patients had neurologic abnormalities. WES facilitated rapid diagnosis in 50 % of the patients, especially those with mitochondrial disorders. Yet, in most cases extensive investigation was required after the results were available. Most patients with confirmed molecular diagnoses had autosomal recessive disorders and were homozygous for the rare alleles. Most patients with autosomal dominant disorders and all patients with X-linked disorders had *de novo* mutations. WES results were negative (no pathogenic variants related to patient phenotype were identified) in six patients and incorrect in two patients. One patient had a reported “deleterious” hemizygous mutation in *SLC35A2*, c.617_620del (p.Q206fs), suggesting ‘congenital disorder of glycosylation, TYPE IIm’, but glycosylation studies were normal and healthy brothers had the same mutation. Another patient had “pathogenic” mutation in *MCCC2*, c.1015G > A (p.V339M), but urine organic acids was normal. WES confirmed inborn errors of metabolism (five mitochondrial diseases, three lysosomal storage diseases, and six other disorders) in 14 patients and genetic disorders (14 neurological diseases and three non-neurological diseases) in 17 patients. Variants of unknown significance were identified in 48 patients; 12 had “confirmed pathologic variants”and 12 had “likely pathologic variants”, based on consistent phenotypes, biochemical/ segregation studies, or reported pathogenicity. In 24 patients, the variants were inconsistent with phenotypes or biochemical/ familial studies.

**Conclusions:**

Although WES provided molecular diagnoses, the results required careful interpretations and many patients required additional investigations. This tool is useful when conventional diagnostic methods fail. Staff competence in obtaining consent/ permission, interpreting the findings, and providing the proper counseling are essential before incorporating this technology into routine clinical practices.

## Background

Emirati people (citizens of United Arab Emirates, UAE) have diverse ethnicities that include lineages from the Arabian Peninsula, Persia, Baluchistan, and East Africa. The culture is primarily tribal and encourages intra-tribal (consanguineous) marriages [1]. Thus, “founder” mutations are prevalent, which markedly increase the frequency of autosomal recessive disorders [2]. Consistently, the prevalence of inborn errors of metabolism (IEM) in the region is high (about 1 in 1,329) [1]. Genetic disorders are also exceptionally common [3].

Diagnosing IEM is often challenging due to their genetic heterogeneity and atypical or overlapping phenotypes. Using clinical assessments and conventional investigations (having an estimated cost of about $25,000 per patient), the rate of molecular diagnosis in these patients is usually less than 50 % [4]. In these families, the lack of a diagnosis poses significant consequences, such as patients missing treatments and failure by medical staff to provide proper counseling and guidance.

The relatively new term “exome” refers to all exons in the human genome. The term “exons” refers to the ~180,000 genomic sequences, which transcribe and remain in the mature RNA. Exome constitutes only 3 % of the human genome and represents the ~22,000 protein-coding genes, mutations of which are responsible for about 85 % of our significant clinical diseases [5–7]. Therefore, sequencing of the exome is thought to be an efficient method of analyzing a patient's DNA to discover the genetic cause of disease. WES is especially efficient in detecting rare mutations in autosomal recessive diseases in consanguineous families.

The recently introduced WES technology offers an adjunctive analytical tool to locus-specific and gene-panel testing for patients with wide-spectrum phenotypes. This study describes diagnostic rate, advantages, and limitations of WES in the UAE.

## Methods

This study was approved by Al-Ain Medical Human Research Ethics Committee (protocol number 10/09). Between January 2012 and December 2014, at Tawam Hospital (Al Ain, Abu Dhabi), 85 WES studies were requested. The risks and benefits of the test were explained to families and patients and written informed consent/ permission was obtained. WES was requested because of atypical phenotypes, overlapping features, or negative biochemical studies.

Blood (5 mL in EDTA-Vacutainer) was obtained from patients, parents and affected family members (when applicable). Extracted DNA was shipped to Whole Genome Laboratory (WGL) of Baylor College of Medicine (Houston, TX, USA). Single WES was performed at WGL as previously reported [6]. Briefly, genomic DNA from patients was fragmented by sonication. The fragments were ligated to illumina multiplexing paired-end adapters, amplified by polymerase chain-reaction assay, and hybridized to biotin-labeled VCRome (at 47 °C for 64–72 h). Paired-end sequencing was performed on IlluminaHiSeq 2000 platform. The mean coverage of the exome was 100 – 120X; and 95 % of the exome was covered at >20X coverage. The output data from IlluminaHiSeqwere converted from bcl file to FastQ file by Illumina Consensus Assessment of Sequence and Variation Software and mapped by Burrows-Wheeler Aligner (BWA) program. Variants were filtered using stringencies of minor allele frequencies and mutation databases and disease specific databases. The results (with disease phenotype) were interpreted by molecular laboratory and medical directors certified by American board of Medical Genetics. Variant annotation and filtering were performed using Atlas-SNP and Atlas-indel.16, in addition to CASSANDRA software. Variants with a minor allele frequency of less than 5 % (according to either the 1000 Genomes Project18 or the ESP5400 data of the National Heart, Lung, and Blood Institute GO Exome Sequencing Project [http://evs.gs.washington.edu/EVS]) were retained. Variants were interpreted according to American College of Medical Genetics guidelines and patient phenotypes. Variants related to patient phenotypes were confirmed by Sanger sequencing for the patients, parents and affected relative if applicable. The interpretation of genes related to the patient’s clinical phenotype was based on the clinical information of the patient. WES reports included also In silico-predictions for non-synonymous (missense) changes by SIFT and PolyPhen-2 [6]. Table 1 lists variants of unknown significance that confirmed the clinical disorder after further investigations. Tables 2, 3 list pathogenic mutations that directly confirmed the clinical disorder without a need for further investigation.

**Table 1:** Variants of unknown significance, which were confirmed pathogenic or likely pathogenic

*Group A: Confirmed pathogenic (n = 12)* ^*a*^			
Genes (Isoforms)	Variants (PMID)	Diagnoses (MIM)	Comments
Autosomal Recessive Inheritance			
*TALDO1* (NM_006755)	c.574C > T (p.A192C) (23315216)	Transaldolase deficiency (606003)	Elevated level of polyol in urine. Affected family members had the same variant.
*ETHE1* (NM_014297)	c.488G > A (p.R163Q) ^b^(14732903)	EE (602473)	Urine organic acid and acylglycine profiles were consistent with ethylmalonic encepathopathy.
*FBXL4 c*	c.1304G > T (p.R435L) ^b^(23993193)	MTDPS13(615471)	Affected sibling had the same variant and healthy siblings were negative.
*DGUOK* (NM_080916)	c.763_765del (p.D255del) ^b^	MTDPS3 (251880)	Affected sibling had the same variant.
*PEX16* (NM_004813)	c.859C > T (p.R287C) ^b^	PBD8B (614877)	Elevated level of very long chain fatty acids, low C26 beta-oxidation in plasma and fibroblasts. Fibroblasts showed enlarged peroxisomes. Affected sibling had the same variant.
*HEXB* (NM_000521)	c.272G > C (p.C91S) ^b^	Sandhoff disease (268800)	Low serum hexosaminidase activity. A pathologic variant in *MCCC2* [c.1015G > A (p.V339M)] was also found, but urine organic acids were normal (PMID: 11181649).
*ATP8B1* (NM_005603)	c.379C > G (p.L127V)^b^	BRIC (243300)	Affected sibling had the same variant and healthy siblings were negative.
*MTPAP* (NM_018109)	c.1468G > T (p.V490L)	SPAX4 (613672)	Developmental delay and regression at 8 months of age, central hypotonia, short stature, failure to thrive, cerebellar atrophy, absence-like episodes, and hip dislocation. Affected sibling had the same variant. Parents were heterozygous.
*RNASEH2C* (NM_032193)	c.205C>T (p.R69W) (16845400)	AGS3 (610329)	Global developmental delay, central hypotonia, peripheral hypertonia, opisthotonus, microcephaly, failure to thrive, diffuse white matter hyperintensity, cortical brain atrophy, and dilated ventricles. WES also reported homozygous variant in *CDK5RAP2* [c.412G > A (p.G138S); an unaffected sibling was homozygous for the same variant] and pathogenic heterozygous mutation in *BUB1B* [c.2441G > A (p.R814H)].
Autosomal Dominant Inheritance			
*FOXG1*	***c.1397G > A (p.G466E)*** ^b^	Rett (613454)	Developmental regression, hypotonia, failure to thrive.
*ANK2* (NM_001148)	*c.1135C > T (p.R379C)*	Long QT (600919)	Two siblings died of cardiac arrest; affected sibling had the same variant. Parents were heterozygous, but their phenotype was not investigated.
X-Linked Inheritance			
*CDKL5* (NM_003159)	***c.593G=/>A (p.G198D)***	EIEE2 (300672)	Global developmental delay and regression, intellectual disability, hypertonia, and seizure disorder. Parents were negative.
*Group B: Likely pathogenic (n = 12)* ^a^			
Autosomal Recessive Inheritance			
*SLC4A4* (NM_000334)	c.2230G > A (p.A744T)	RTA, proximal (604278)	Severe RTA and ocular hypertension. Parents were heterozygous.
*G6PC* (NM_000151)	c.352G > C (p.A118P) (24980439)	GSD1A (232200)	Hypoglycemia, hepatosplenomegaly, ↑lactate, left ventricular hypertrophy, and family history of cardiomyopathy.
*MPDZ* (NM_003829)	*c*.[394G>A]; [1744C>G] *p.[G132S); [L582V]*	HYC2 (615219)	Communicating hydrocephaly.
*TTN* (NM_133378)	*c.[9160 G > C;68120 A > G]; [74633C > T] p.[E3054;Y22707C];[A24878V]*	CMD1G (604145	Mother was asymptomatic with the heterozygous c.9160 G > C and c.68120 A > G. Father was heterozygous for c.74633C > T.
*MIPEP* (NM_005932)	c.1027A > G (p.K343E)	MIPEP (602241)	Developmental delay, hypotonia, dysmorphism, microcephaly, vision loss, and atrial septum defect.
*ALG9* (NM_024740)	c.694G > C (p.A232P)	CDG1L (608776)	Patient had global developmental delay and regression, central and peripheral hypertonia, seizures, macrocephaly, brain atrophy, thin corpus callosum, and cysts in the white matter. WES also reported homozygous variants in *NDUFV3* [c.552dup (p.L185fs)] and in *KANK1* [c.1079G > A (p.S360N)].
*GLRA1* (NM_000171)	c.1214G > A (p.R405Q)	HKPX1 (149400)	Global developmental delay, hypotonia then hypertonia, seizures, repetitive hand movements, startle reflex to tactile and sound, dysmorphic features (elongated face, big prominent ears), tall habitus, squint, scoliosis, and precious puberty. WES also reported homozygous variants in *TTC37* [c.1828A > G (p.S610G)] and in *CHD8* [c.1952G > A (p.R651Q)].
*PLA2G6* (UC003aux.1)	c.154G > A (p.V52M)	NBIA2B (610217)	Developmental regression, hypertonia, failure to thrive, scoliosis, and skin anomalies. Brain MRI findings were consistent with NBIA2B. Cousin with spina bifida and hydrocephalus. WES also reported homozygous variant in *ACOX1* [c.1165A > G (p.I1389V)] and compound heterozygous variant in *SACS* [c.2143C > A (p.P715T) and c.5732C > A (p.T1911M)].
Autosomal Dominant Inheritance			
*ABCB6* (NM_005689)	*c.[4G > A; 904C > G] p.[V2M;L302V]*	MCOPCB7 (614497)	Autism, subtle microphthalmia, and repetitive hand movements. WES also identified a heterozygous variant in *CHD7* [*c.5689G > A (p.E1897K)*]*.* (CHARGE, MIM: 214800) Father had small eyes and was heterozygous for both changes.
*ITPR1* (NM_002222)	*c.3758 T > A (p.I1253N)*	SCA15 (606658)	Ataxia and cerebellar atrophy without deafness. WES also reported a variant in *MYH14* [c.1126G > T (p.G376C)]. (PMID: 15015131, MIM: 600652)
*CHRNB1* (NM_000747)	***c.865G > A (p.V289M)*** (8872460)	CMS2A (616313)	Delayed motor milestone, hypotonia, pulmonary hypertension, seizures, and autistic features. Nerve conductive study was normal. Father had paranoid schizophrenia. This variant was previously reported (PMID:8872460).
*KIF5C* (NM_004522)	***c.404A > G (p.Y135C)***	Cortical dysplasia, complex, with other brain malformations 2 (615282)	The disease is autosomal dominant. All family members were negative for the variant. WES also reported a variant in *NRXN1* [c.1835A > G (p.D612G)]. Parents and one sibling were heterozygous (MIM: 614325).

^a^ Group A was based on consistent phenotypes, biochemical findings, familial (segregation) studies, or reported pathogenicity

^**b**^ Novel mutations

Mutations in bold were *de novo*

All mutations were homozygous, except those in *Italics* which were heterozygous

^c^ WES was done in Netherland

*PMID* PubMed Identifier, *MIM* Mendelian Inheritance in Man, *EE* encephalopathy, ethylmalonic, *MTDPS13* mitochondrial DNA depletion syndrome 13 (encephalomyopathic type), *MTDPS3* mitochondrial DNA depletion syndrome 3 (hepatocerebral type), *PBD8B* peroxisome biogenesis disorder 8B, *BRIC* cholestasis, benign recurrent intrahepatic, *SPAX4* spastic ataxia 4, autosomal recessive, *AGS3* aicardi-goutieres syndrome 3, *Long QT* cardiac arrhythmia, ankyrin-B-related, *EIEE2* epileptic encephalopathy, early infantile

^a^ Group B was based on consistent phenotypes or previously reported probable pathogenicity

All mutations were homozygous, except those in Italics which were heterozygous

*RTA*, renal tubular acidosis, *GSD1A* glycogen storage disease Ia, *HYC2* hydrocephalus, nonsyndromic, autosomal recessive 2, *CRS3* craniosynostosis 3, *CMD1G* cardiomyopathy, dilated, 1G, *MIPEP* mitochondrial intermediate peptidase, *CHARGE* CHARGE syndrome, *CDG1L* congenital disorder of glycosylation, type lL, *HKPX1* hyperekplexia, hereditary 1, *NBIA2B* neurodegeneration with brain iron accumulation 2b, *MCOPCB7* microphthalmia, isolated, with coloboma 7, *SCA15* spinocerebellar ataxia 15, *CMS2A* myasthenic syndrome, congenital, 2a, slow-channel

**Table 2:** Confirmed IEM by WES. *n* = 14

Gene (Isoform)	Variants (PMID)	Diagnosis (MIM)	Comments
Mitochondrial inheritance			
*tRNA Ala*	m.5591G > A^b^ (16476954)	MTTA (590000)	Failure to thrive, ptosis, myopathy, normal mitochondrial studies on muscle biopsy.
*ND5*	m.13513G > A (p.D393N)^b^ (12509858)	MTND5 (516005)	Developmental regression, dysmorphic features, esotropia, chorioretinal atrophy, seizure, left ventricular hypertrophy, renal insufficiency, brain image suggestive of Leigh syndrome.
X-Linked inheritance			
*PDHA1* (NM_000284)	*c.787C > G (p.R263G)* (1508605)	PDHAD (312170)	Brain image suggestive of Leigh syndrome; low complex II (succinate dehydrogenase) activity in the fibroblasts. Two heterozygous (‘*cis*’ configuration) novel missense variants, c.321C > G (p.1107 M) and c.338A > T (p.N113I), in *SDHA* were found in patient and father.
Autosomal recessive inheritance			
*FBXL4* (NM_012160)	c.1067delG (p.G356fs) (23993194)	MTDPS13 (615471)	Developmental regression, hypotonia, failure to thrive, microcephaly, lactic acidosis, normal fibroblast mitochondrial studies. Three siblings died in infancy.
*C10orf2* (NM_021830)	c.1198C > T (p.R400C) (21364701)	MTDPS7 (271245)	Developmental regression, hearing loss, scoliosis, reduced activities of complexes I & IV in myocytes; normal activities in fibroblasts. Two cousins with Leigh disease.
*MTHFR* (NM_005957)	c.1596C > G(p.Y532) ^(a)^	MTHFRD (236250)	Progressive encephalopathy, seizure, cerebral venous thrombosis, gangrenous like bullous formation in the leg, congenital heart disease, ↑homocysteine, ↓methionine.
*PYCR2* (NM_013328)	c.28C > T(p.Q10X) ^(a)^	HLD10 (616420)	Developmental regression, failure to thrive, seizure, microcephaly, severe demyelination, thin corpus callosum.
	c.796C > T(p.R266X) ^(a)^		Developmental delay, hypotonia, failure to thrive, microcephaly, thin corpus callosum, delayed myelination.
*HEXA* (NM_000520)	c.2 T > C	Tay-Sachs (268800)	Developmental regression, failure to thrive, seizure disorders, dystonia, feeding difficulties constipation. Diagnosis confirmed by enzyme analysis.
*HEXB* (NM_000521)	c.826_829del (p.E276fs)	Sandhoff (268800)	Developmental regression, failure to thrive, feeding difficulties, seizure, and vision loss.
*SUMF1* (NM_182760)	*c.691dupT (p.W231fs) c.689A > G (p.E230ZG)*	MSD (272200)	Developmental delay, seizure, hepatomegaly, delayed myelination, ↑urine sulfatide, ↑urine heparan sulphate.
*UROC1* (NM_144639)	c.855G > A (p.W285X) ^(a)^	UROCD (276880)	Intellectual disabilities, attention deficit and hyperactivity disorder, hyperextensible joints, ↑imidazole propionate.
*TBX19* (NM_005149)	c.604-1G > C ^(a)^	IAD (201400)	Intellectual disabilities, congenital hypothyroidism, two sisters died in infancy with hypoglycemia.
*HSD3B7* (NM_025193)	c.45_46del (p.G17fs) (12679481)	CBAS1 (607765)	Neonatal cholestasis, hepatosplenomegaly, hypotonia, failure to thrive.

^(a)^ Novel mutation; (b) heteroplasmic mutations (56-59 %). Mutations in bold are *de novo*, All mutations are homozygous, except those in Italics, which are heterozygous. *PMID* PubMed Identifier, *MIM* Mendelian Inheritance in Man, *MTTA* transfer RNA, mitochondrial, alanine, *MTND5* complex I, subunit ND5, *PDHAD* pyruvate dehydrogenase e1-alpha deficiency, *SDHA* succinate dehydrogenase complex, subunit A, flavoprotein, *MTDPS13* mitochondrial DNA depletion syndrome 13 (encephalomyopathic type), *MTDPS7* mitochondrial DNA depletion syndrome 7 (hepatocerebral type); *MTHFRD* methylenetetrahydrofolate reductase deficiency, *HLD10* leukodystrophy, hypomyelinating 10, *MSD* multiple sulfatase deficiency, *UROCD* urocanase deficiency, *IAD* ACTH deficiency, *CBAS1* bile acid synthesis defect, congenital, 1

**Table 3:** Confirmed genetic diseases by WES, *n* = 17

Gene (Isoform)	Variants (PMID)	Diagnosis (MIM)	Comments
X-Linked Inheritance			
*PAK3* (NM_002578)	*c.1279 T > C (p.Y427H)*	MRX30 (300558)	Intellectual disabilities, macrocephaly, obesity.
Autosomal Dominant Inheritance			
*BRAF* (NM_004333)	*c.1914 T > G (p.D638E)* (19206169)	CFC1 (115150)	Cortical blindness, seizures, stridor, constipation and developmental delay.
*DYNC1H1* (NM_001376)	*c.10973G > A (p.G3658E)*	MRD13 (614563)	Developmental regression, seizure, microcephaly, cataract, lissencephaly, pachygyria, grey matter heterotopia, hypoplasia of the corpus callosum.
*ARID1B* (NM_020732)	*c.4870C > T (p.R1624X)* ^(a)^	Coffin-Siris (135900)	Mucopolysaccharidosis suspected clinically.
*ARID1B* (NM_020732)	*c.3689 + 1G > C*	Coffin-Siris (135900)	Global developmental delay, failure to thrive, acute encephalopathy with hypoglycemia and metabolic acidosis.
*MYBPC3* (NM_000256)	*c.776delinsTT (p.A259fs)*	CMD1MM (615396)	Dilated cardiomyopathy.
Autosomal Recessive Inheritance			
*SNX10* (NM_001199835)	c.112-1G > C	OPTB8 (615085)	Central hypotonia, optic atrophy, osteopetrosis, pulmonary hypoplasia, hyperpigmented macules.
*TRAPPC11* (NM_199053)	c.2938G > A (p.G980R) (23830518)	LGMD2S (615356)	Developmental delay, head nodding, hypotonia, ↑CPK, ↑plasma phenylalanine, normal CSF neurotransmitters. Homozygous c.362 T > C (p.I121T) variant in *COQ9* (normal muscle coenzyme Q10 activity).
*PCSK1* (NM_000439)	c.1312C > T (p.R438X)	Proprotein convertase 1/3 deficiency (600955)	Brain hemorrhage, congenital diarrhea.
*ERCC5* (NM_000123)	c.205C > T (p.R69X)^(a)^	Cockayne (278780)	Hypotonia, developmental delay and seizure. Clinically suspected to have MLCD.
*AHI1* (NM_017651)	c.1051C > T (p.R351X) (15322546)	Joubert syndrome-3 (608629)	Intellectual disability, hypotonia, repetitive hand movements, brain atrophy.
*PRX* (NM_181882)	c.1090C > T (p.R364X) (21741241)	Dejerine-Sottas (145900)	Abnormal gait, hearing loss, loss of dexterity in hands, scoliosis.
*TREX1* (NM_003629)	c.341G > A (p.R114H) (21270825)	RVCL (192315)	Cognitive impairment, hypotonia, joint contracture, glaucoma, brain atrophy, sibling died with the same features.
*DOK7* (NM_173660)	*c.1124_1127dup (p.A378fs) & c.1457dup (p.A487fs)* (16917026)	Myasthenia, limb-girdle (254300)	Hypotonia, myopathic changes in proximal muscles.
*ADD3* (NM_016824)	c.1100G > A (p.G367D) (23836506)	Adducin-gamma (601568)	Developmental delay, central hypotonia and peripheral spasticity, cortical brain atrophy, delayed myelination of white matter.
*RECQL4* (NM_004260)	c.1000G > T (p.E334X)^(a)^	RTS (268400)	Premature, intrauterine growth retardation, dry skin, clinically suspected to have MOPD2.
*IKBKB* (NM_001556)	c.849G > A (p.W283X)^(a)^	IMD15 (615592)	Failure to thrive, recurrent infections, two sibling died with the same presentation.

^(a)^ Novel mutation. All mutations are homozygous, except those in *Italics* which are heterozygous. Variants in bold are *de novo* pathologic mutations. *PMID*,PubMed Identifier, *MIM* Mendelian Inheritance in Man, *MRX30* mental retardation, X-linked 30, *CFC1* cardiofaciocutaneous syndrome 1, *MRD13* mental retardation, autosomal dominant 13, *CMD1MM* cardiomyopathy, dilated, *1MM; OPTB8* osteopetrosis, autosomal recessive 8; *LGMD2S* muscular dystrophy, limb-girdle, type 2S, *MLCD* microcephaly-lymphedema chorioretinal dysplasia syndrome, *RVCL* vasculopathy, retinal, with cerebral leukodystrophy; isolated, *RTS* Rothmund-Thomson syndrome, *MOPD2*, Microcephalic osteodysplastic primordial dwarfism, *type I*I, *IMD15* Immunodeficiency 15

## Results

Eighty-five patients (46 % females) had WES testing. WES results were divided into the following categories: [1] Variants of unknown significance, which were confirmed pathogenic (12 patients, Group A) or likely pathogenic (12 patients, Group B) based on consistent phenotypes, biochemical findings, familial (segregation) studies, or reported pathogenicity (Table 1); [2] WES diagnosis of IEM (14 patients, Table 2); [3] WES diagnosis of genetic diseases (17 patients, Table 3); [4] Variants of unknown significance, which were inconsistent with patients’ phenotypes or biochemical/ familial (segregation) studies (24 patients); and [5] No pathogenic variants related to patient phenotype were identified (six patients).

Patients who had variants of unknown significance were investigated using biochemical tests, familial (segregation) studies, and a review of the literature. Patients with consistent phenotypes, biochemical findings, familial (segregation) studies, or reported pathogenicity were considered “confirmed pathogenic” (Table 1A). Patients with consistent phenotypes or previously reported probable pathogenicity were considered “likely pathogenic” (Table 1B). The 24 patients who had ‘variants of unknown significance’ that were inconsistent with their phenotypes or biochemical/ familial (segregation) studies were not included in this report.

### A. Patients’ demographics

Eighty of the 85 (93 %) patients were younger than 18 years and 44 (52 %) were younger than 5 years. Sixty-eight (80 %) patients had neurologic abnormalities. Patients had appropriate metabolic and genetic investigations (e.g., metabolic screening, microarray analysis, skin/ muscle biopsy, biochemical studies, and DNA testing) before WES testing. For most of patients, both parents DNA were tested. The cost of WES was billed to insurance companies in the UAE.

### B. Molecular diagnoses

Fourteen (16 %) patients had IEM (Table 2), 17 (20 %) had genetic syndromes (Table 3), 12 (14 %) had variants of unknown significance confirmed to be pathogenic (Table 1A), and 12 (14 %) had variants of unknown significance likely to be pathogenic (Table 1B). Therefore, WES information was clinically useful in 55 (65 %) patients and diagnostic in 43 (50 %) patients.

Forty-one (75 %) of the 55 patients had consanguineous families. All patients with autosomal recessive diseases had consanguineous families (Tables 1–3); except the patient with *MTHFR* mutation and the patient with *TBX19* mutation (Table 2) who had families of the same tribe/ region; the patient with compound heterozygous mutations in *MPDZ* (Table 1) and the patients with *PCSK1, AHI1,* and *DOCK7* mutations (Table 3). The patients with autosomal dominant mutation in *CHRNB1* and *FOXG1* (Table 1), the patient with mitochondrial mutation in *tRNA Ala* (Table 2), and the patient with X-linked mutation in *PDHA1* (Table 2) had non-consanguineous families. In Table 3, the patients with X-linked or autosomal dominant conditions had non-consanguineous families, except for the patient with *MYBPC3* mutations.

One hundred twenty-eight variants were identified. Forty-four (34 %) variants were pathologic. Twenty (15 %) of these variants were previously reported and 24 (19 %) were novel.

### C. Autosomal dominant mutations

Eleven (13 %) patients had autosomal dominant disorders. Five patients had truncating or missense mutations which were previously reported. Three of these mutations were *de novo* (Table 3). Six patients had autosomal dominant (Table 1); three were *de novo* variant, one was inherited from symptomatic parent, and two were inherited from parents of unknown phenotype.

### D. Autosomal recessive mutations

Forty (47 %) patients had autosomal recessive diseases. Thirty-five patients had homozygous variants and five patients had compound heterozygous variants. Eleven pathogenic mutations involved inborn errors of metabolism (Table 2). Five of these mutations were novel, including a compound heterozygous mutation (Table 2). Eleven pathogenic homozygous mutations involved genetic syndromes (Table 3). Five of these mutations were previously reported and six were novel (Table 3).

Of the reported variants of unknown significance, nine were confirmed pathogenic homozygous mutations on further testing, six of which were novel (Table 1A). Seven of these disorders were IEM (Table 1A). In addition, six homozygous and three compound heterozygous variants were likely pathogenic; only one of these variants was previously reported (Table 1B).

### E. X-linked mutations

The three patients with X-linked disorders had *de novo* mutations; one had pyruvate dehydrogenase e1-alpha deficiency (Table 2) and one had mental retardation, X-linked 30 (Table 3). The patient with epileptic encephalopathy, early infantile had mosaic *de novo* variant (Table 1A).

### F. Mitochondrial mutations

Two female patients had known pathogenic mutations. The patient with Leigh disease had 56 % heteroplasmic m.13513G > A (p. D393N, ND5) mutation in the mitochondrial *ND5* gene. The second patient had mitochondrial myopathy with 59 % heteroplasmic m.5591G > A (tRNAAla), Table 2.

### G. Carrier status

Carrier status for Mendelian recessive disorders was detected in nine patients. Two patients had sickle cell trait, two had β-thalassemia trait, one had *CFTR* gene mutation (cystic fibrosis heterozygosity), and four females had *G6PD* gene mutations (glucose-6-phosphatase dehydrogenase heterozygosity).

### H. Medically actionable genetic variants

Medically actionable genetic variants unrelated to the clinical phenotype were identified in eight patients. Seven patients had mutations in the *G6PD* gene (c.563C > T in five patients, c.1003G > A in one patient, andc.634A > G in one patient) and one had mutation in the *MYBPC3* gene (c.2148 + 1G > A) that causes dilated cardiomyopathy.

## Discussion

In this study, WES provided useful clinical information in most patients in whom several other tests were not revealing. It attained a diagnostic rate of 50 % compared with25–42%in other studies [6–11]. This higher yield is a result of the frequent autosomal recessive diseases (mostly homozygous mutations) in the region and the multiple affected children in the same family.

Thirty-one of the 43 (72 %) patients with confirmed molecular diagnoses had autosomal recessive disorders compared with 34 % in another study [8]. Twenty-nine of these patients were homozygous for the rare allele, indicating consanguinity played a major role in making autosomal recessive disorders frequent in this culture.

Of the patients with autosomal dominant disorders, 66 % had *de novo* mutations (Table 1 and Table 3). In addition, all patients with X-linked disorders had *de novo* mutations (Table 1A and Tables 2, 3). WES detected a low-level mosaicism in one patient with X-linked *de novo* variant in *CDKL5,* which was consistent with his phenotype (Table 1). WES identified a mutation in *G6PC* causing GSD1a (Table 1) in a patient who was clinically suspected to have GSD III or IX due to cardiomyopathy and hepatic manifestations but had normal leukocyte enzymes for GSD III and IX. Establishing GSD1a diagnosis in this patient enabled appropriate counseling and genetic screening of the family members.

WES was especially helpful in a few patients with suspected mitochondrial disorders in whom extensive mitochondrial work-up was not revealing. Mutations in *FBXL4* and *C10orf2* causing mitochondrial DNA depletion syndrome were identified (Table 2). WES facilitated a rapid diagnosis in these patients with mitochondrial disorders, avoiding a need for invasive muscle or skin biopsies.

WES also provided a relatively rapid diagnosis in patients with treatable conditions, such as glycogen storage diseases (Table 1B). In addition, it provided a diagnosis in patients with atypical features of rare diseases, such as the patient with global developmental delay, repetitive hand movements, and brain atrophy without the molar tooth sign. This patient was confirmed to have Joubert syndrome due to a homozygous mutation in *AHI1,* c.1051C > T (p.R351X) (Table 3). In addition, WES revealed pathological variants in *ARID1B* in two patients (Table 3); one of them was suspected to have mucopolysaccridosis. Founder mutations were identified in this tribal population, such as the pathologic variant causing transaldolase deficiency (Table 1). This finding facilitated further diagnoses in our community using single gene sequencing.

It is important to note that the results of WES should be appraised carefully. In one patient, WES reported a deleterious hemizygous mutation in *SLC35A2,* c.617_620del (p.Q206fs),congenital disorder of glycosylation, TYPE IIm (CDG2M). However, glycosylation screening was normal in this patient and three healthy brothers had the same mutation (not listed in the table). In another patient, WES reported a pathogenic mutation in *MCCC2*, c.1015G > A (p.V339M), but urine organic acids were normal and the patient had Sandhoff disease (confirmed by enzyme studies), Table 1.

In most cases, extensive and expensive investigations (including biochemical, genetic, and segregation studies) were necessary after the results of WES to determine the pathogenicity of the variants. In UAE, most families are consanguineous and have multiple affected individuals. Therefore, it was somewhat possible to stratify many of the variants of unknown significance as pathogenic *vs* non-pathogenic. Nevertheless, challenges remain in the interpretation of the 12 likely pathogenic variants (Table 1b). The availabilities of functional studies, improved knowledge of rare allele frequencies of healthy individuals, and more routine use of WES will improve the yield of this powerful analytical tool.

Competent staff is needed to explain WES results and to counsel patients and their families. The metabolic centers should develop and adhere to appropriate guidelines for utilizing WES as a diagnostic tool. Position statements and best practice guidelines are available from the American college of Medical Genetics, Canadian college of Medical Genetics and European society of Human Genetics [12–14].

Many variants are confined to ethnic populations and are, thus, difficult to diagnose by conventional genetic methods [2, 15]. The cost of diagnosing genetic disorders using conventional testing was estimated to be about $25,000 per patient [4]. However, it is unknown whether WES would increase or decrease this cost. In this study, the cost of WES was covered by local insurance companies for UAE citizens; the cost is a critical issue for the majority of patients who are not citizens of this country.

## Conclusion

In this study, with the majority of patients had consanguineous families, WES identified pathogenic mutations in 50 % of cases. WES also provided population-specific information, which should facilitate molecular diagnosis of metabolic and genetic disorders in the region. However, further studies, including cost-effective assessments are needed before routine application of this test in IEM and genetic clinics.

## Abbreviations

BWA, burrows-wheeler aligner; CDG2M, congenital disorder of glycosylation, TYPE IIm; CFTR, cystic fibrosis transmembrane conductance regulator; DNA, deoxyribonucleic acid; EDTA, ethylenediaminetetraacetic acid; G6PD, glucose-6-phosphatase dehydrogenase; GSD1A, glycogen storage disease Ia; IEM, inborn errors of Metabolism; MIM, mendelian Inheritance in man; RNA, ribonucleic acid; SNP, single nucleotide polymorphism; TX, Texas; UAE, United Arab Emirates; USA, United States of America; WES, whole exome sequencing; WGL, whole genome laboratory
